# The Presence of Previous Cerebral Microbleeds Has a Negative Effect on Hypertensive Intracerebral Hemorrhage Recovery

**DOI:** 10.3389/fnagi.2017.00049

**Published:** 2017-03-07

**Authors:** Kang Yang, Yulan Feng, JinJin Mu, Ningzhen Fu, Shufen Chen, Yi Fu

**Affiliations:** ^1^Department of Neurology and Institute of Neurology, Rui Jin Hospital, School of Medicine, Shanghai Jiao Tong UniversityShanghai, China; ^2^Department of Neurology, Min Hang Hospital, Fudan UniversityShanghai, China; ^3^School of Medicine, Shanghai Jiao Tong UniversityShanghai, China

**Keywords:** cerebral microbleeds, silent brain infarction, hypertension, intracerebral hemorrhage, magnetic resonance imaging

## Abstract

**Background and Purpose:** Cerebral microbleeds are an intracerebral microangiopathy with bleeding tendency found in intracerebral hemorrhage patients. However, studies about cerebral microbleed effects on the prognosis of hypertensive intracerebral hemorrhage patients are rare. We performed a prospective study to discuss not only the risk factors of cerebral microbleed incidence in hypertensive intracerebral hemorrhage patients but also the relevance of cerebral microbleeds with silent brain infarction, hemorrhage and prognosis.

**Methods:** This study enrolled 100 patients diagnosed with hypertensive intracerebral hemorrhage within 3 days after onset. Magnetic resonance imaging including susceptibility-weighted imaging and diffusion-weighted imaging (DWI) were utilized to examine patients on the fifth day after onset. Regular follow-ups were performed to examine the following clinical cerebrovascular events and vascular deaths in 1 year.

**Results:** Cerebral microbleeds were observed in 55 (55%) patients. Multiple logistic regression analysis showed that over-aging, elevation of serum creatinine, and leukoaraiosis were independently associated with cerebral microbleeds. In addition, higher silent brain infarction prevalence was observed in patients with cerebral microbleeds. In contrast, none of the cerebral microbleed patients exhibited cerebral microbleeds ≥5, which is an independent risk factor of poor 3-month neurological function recovery. During the 1-year follow-up, 14 subjects presented clinical cerebrovascular events or vascular death. The Cox proportional hazards model implicated that atrial fibrillation, cerebral microbleeds ≥5 and silent brain infarction were independent predictive factors for these events.

**Conclusions:** Over-aging combined with an elevation of serum creatinine and leukoaraiosis were independent risk factors of cerebral microbleeds. Patients with cerebral microbleeds were more likely to exhibit silent brain infarction. Poor recovery of 3-month neurological function was observed in hypertensive intracerebral hemorrhage patients with cerebral microbleeds ≥5. Cerebral microbleeds ≥5 or silent brain infarction might also indicate an elevated risk of future cerebrovascular events and vascular death.

## Introduction

Spontaneous intracerebral hemorrhage occurs at an incidence of 10~30% of first-ever strokes, and 30~55% of patients reach mortality in 1 month (Balami and Buchan, 2012; Tsai et al., 2013). However, hypertensive intracerebral hemorrhage (HICH) occurs in 70~80% of spontaneous intracerebral hemorrhages (ICHs). In addition, the incidence of CMBs in ICH patients can reach 50~80% (Greenberg et al., 2004; Jeong et al., 2004; Imaizumi et al., 2008), which is much higher than the 15~35% observed in cerebral infarction patients (Fiehler et al., 2007; Yamada et al., 2012). CMBs are the haemosiderin exuded from cerebral small vessels and precipitated around small vessels. The characteristics of CMBs in gradient echo T2^*^-weighted imaging (GRE T2^*^WI) or susceptibility-weighted imaging (SWI) are small circumscribed round foci and low signals (Greenberg et al., 2009b). Furthermore, CMBs occurring in the deep brain are mostly related to small vessel diseases induced by atherosclerosis or hypertension, while CMBs focused in lobes are more likely caused by cerebral amyloid angiopathy (Fazekas et al., 1999; Knudsen et al., 2001).

Unfortunately, few studies targeting the effect of CMBs on the prognosis of HICH patients have been performed. Moreover, the subjects are mainly acute cerebral infarction patients (Dannenberg et al., 2014; Gratz et al., 2014; Turc et al., 2015; Yan et al., 2015). Nevertheless, an increasing number of scholars have realized these challenges and are working to resolve them. Due to the report indicating that the risks of disabling and fatal strokes could increase in acute cerebral infarction patients or transient ischaemic attack with CMBs (Boulanger et al., 2006), we aimed to determine whether CMBs could increase these risks in HICH patients. Previous studies have revealed that the GRE T2^*^WI sequence is broadly used in CMBs research studies (Lee et al., 2006; Kang et al., 2012; Turc et al., 2015). However, recent studies have indicated that the SWI sequence demonstrated a better detection rate than the GRE T2^*^WI sequence (Guo et al., 2013). Thus, we utilized SWI to detect CMBs to determine whether CMBs could indicate a higher probability for the incidence of clinical cerebrovascular events or vascular deaths in the following first year, which may clinically benefit HICH patients.

## Methods

### Patients

We prospectively and continuously recruited acute intracerebral hemorrhage patients from August 2012 to April 2015 in the Department of Neurology of the Affiliated Rui Jin Hospital of Shanghai Jiao Tong University and Affiliated Min Hang Hospital of Fudan University. Regular follow-ups were performed during the following year after onset. The inclusion criteria were as follows: (1) age over 35 years; (2) diagnosed as acute intracerebral hemorrhage in 3 days by CT after onset; and (3) possessing hypertension history. The definition of hypertension was as follows: (1) with hypertension history or (2) with hypertension in three separate sphygmomanometer measurements during the admission period (systolic pressure ≥140 mmHg or diastolic pressure ≥90 mmHg) combined with evidence of end-stage organ injury (e.g., hypertensive retinopathy, hypertrophy or enlargement of the left ventricle). The exclusion criteria were as follows: ICH secondary to cerebral tumor; with the possibility to be intracranial hemorrhage related with cerebral amyloid angiopathy (Knudsen et al., 2001); ICH due to abnormal brain structure; moyamoya disease; intracranial ruptured aneurysms; hemorrhage caused by craniocerebral trauma; haematemesis or taking an anticoagulant drug; with contraindications for MRI, pregnant women, or subjects who refused to participate in the study. The time of disease onset was recorded as the last time when the patients were evaluated to be without stroke symptoms. During the admission period, all of the patients received proper medical treatments and supportive care.

This study was approved by the ethics committee of Rui Jin Hospital, which is affiliated with Shanghai Jiao Tong University School of Medicine. All patients or their legal guardian signed the written consent form.

General clinical data, laboratory data and imaging data of the patients were collected as shown in Table 1. The National Institutes of Health stroke scale (NIHSS) and Glasgow Coma Scale (GCS) were selected to evaluate the severity of the neurological functional impairment of the patients upon admission.

**Table 1:** **Univariate analysis of patients with or without CMBs**.

	**CMB not present *n* = 45**	**CMBs present *n* = 55**	***P*-value**
Age, mean (*SD*)	58.0 ± 11.0	62.7 ± 13.5	0.062
Sex, male (%)	28 (62.2%)	37 (67.3%)	0.598
**RISK FACTORS, YES (%)**			
Diabetes mellitus	6 (13.3%)	11 (20.0%)	0.377
Hyperlipidemia	28 (62.2%)	32 (58.2%)	0.682
Current smoking	14 (31.1%)	19 (34.5%)	0.716
Drinking	8 (17.8%)	10 (18.2%)	0.958
Atrial fibrillation	1 (2.2%)	3 (5.5%)	0.758
Prior ischaemic stroke	3 (6.7%)	6 (10.9%)	0.699
**MEDICATIONS, YES (%)**			
Antiplatelet use prior to ICH	3 (6.7%)	13 (23.6%)	0.021
Antidiabetics use prior to ICH	4 (8.9%)	7 (12.7%)	0.773
**ADMISSION LABORATORY DATA (IQR)**			
TG, mmol/L	1.5 (1.2–2.2)	1.5 (1.1–2.0)	0.763
Cholesterol, mmol/L	4.8 (4.0–5.5)	4.7 (4.1–5.4)	0.876
H-DLC, mmol/L	1.1 (0.9–1.3)	1.1 (0.9–1.4)	0.975
L-DLC, mmol/L	3.1 (2.3–3.6)	3.0 (2.5–3.6)	0.771
BUN, mmol/L	4.3 (3.3–5.4)	5.1 (4.1–6.0)	0.038
Cr, umol/L	63.0 (47.5–70.5)	65.0 (54.0–79.0)	0.040
Homocysteine, umol/L	12.1 (9.5–15.8)	16.3 (12.2–22.1)	0.001
Glucose, mmol/L	5.1 (4.6–6.7)	5.0 (4.4–6.6)	0.540
**BLOOD PRESSURE VARIABLES (MMHG)**			
Initial SBP at ER	150.07 ± 21.48	156.65 ± 28.38	0.202
Initial DBP at ER	88.73 ± 11.63	90.07 ± 15.49	0.639
Mean arterial pressure at ER	109.18 ± 14.09	112.27 ± 18.57	0.360
**NIHSS SCORE, MEDIAN (IQR)**			
Baseline	4 (2–6.5)	3 (2–5)	0.057
Discharge	4 (2–5)	3 (2–4)	0.181
Baseline-Discharge	0 (0–2)	1 (0–1)	0.665
GCS	15 (14.5–15.0)	15.0 (15.0–15.0)	0.181
**RADIOLOGIC DATA**			
**ICH location**			
Basal ganglia	25 (55.56%)	27 (49.09%)	
Thalamus	7 (15.56%)	15 (27.27%)	
Pons	3 (6.67%)	3 (5.45%)	
Cerebellum	1 (2.22%)	3 (5.45%)	
Lobar	9 (20%)	7 (12.73%)	
Leukoaraiosis	16 (35.6%)	34 (61.8%)	0.009
Silent brain infarction	1 (2.2%)	10 (18.2%)	0.027

*CMBs, cerebral microbleeds; IQR, interquartile range; TG, triglyceride; BUN, blood urea nitrogen; Cr, creatinine; DBP, diastolic blood pressure; SBP, systolic blood pressure; NIHSS, NIH Stroke Scale; GCS, Glasgow Coma Scale; ICH, intracerebral hemorrhage*.

### Imaging analysis

A cranial CT was performed on all patients on the third day after onset, and a cranial MRI was performed on the fifth day. The GE Signa HDxT 3.0 T superconductor MRI system was selected for MRI examination with eight-channel phased-array head coil utilized for scanning. All patients were first examined with axial T1WI, T2WI and then with diffusion-weighted imaging (DWI) and SWI. The DWI parameters were as follows: single shot echo planar imaging technique; TR/TE = 10,000 ms/Minimum; contiguous layer thickness as 5 mm; spacing as 1 mm; FOV = 230 × 230 mm; matrix as 128 × 64, 128 × 128, and 128 × 256 separately; dispersion sensitivity coefficient (*b*-value) as 0, 1,000 s/mm^2^ separately. The DWI graphs were imported into the GE workstation, and the apparent diffusion coefficient (ADC) graphs could then be calculated using the Functool software. The SWI parameters were as follows: TR/TE = 36/45 ms, flip angle as 20°; contiguous slice thickness as 2 mm; matrix as 448 × 384; NEX = 0.75. All scanning examinations in different medical centers were operated by a skilled radiological technician. Two senior radiologists randomly read and recorded the graphic information. When a conflict occurred, a decision was made upon a final consensus.

The hemorrhage and CMB locations were classified according to anatomical division, including the lobes, basal ganglia, thalamus, brainstem, and cerebellum. The formula used to calculate the cerebral haematoma volumes was the following: (1/2)^*^A^*^B^*^C, in which A referred to the longest diameter of haematoma volumes in the CT axial image, B indicated the short diameter perpendicular to the longest diameter, and C was defined as the product of layers multiplied by contiguous layer thickness (Kazui et al., 1997). Silent brain infarction (SBI) was defined as dispersion abnormal signals more than 20 mm away from the original haematoma area manifesting as low-signal foci in ADC graphs (Wardlaw et al., 2013a). The definition of silent was no new symptoms, such as abnormal sensation and limb weakness or deterioration of the original neurological system function occurring in the patients. However, CMBs were defined as 2~5 mm-diameter round or circular reducing signal shadows and circumscribed round foci with no surrounding oedema (Greenberg et al., 2009a; Wardlaw et al., 2013b). In addition, the signals caused by calcification, perivascular space, small vein, and other factors were excluded. We could distinguish CMBs from HICH using head MRI. First, CMBs were negative in typical T1WI and T2WI sequences and round or oval even at low signal sites in GRE-T2^*^WI and SWI sequences with no surrounding oedema zone (Greenberg et al., 2009a). However, acute phase cerebral hemorrhage induced an equivalent signal in T1WI and a slightly low signal in T2WI, while sub-acute or chronic haematoma showed a high signal in both the T1WI or T2WI. Second, CMBs sites were round or oval with a diameter of 2~5 mm, which were significantly different with the morphology and volume of cerebral hemorrhage haematoma. According to previous research studies, the number of CMBs were then sorted into 4 levels: level 1 with 0 CMBs, level 2 with 1 CMB, level 3 with 2~4 CMBs, and level 4 with more than 5 CMBs (Dannenberg et al., 2014).

### Clinical follow-ups

All patients were provided with proper post-discharge drug therapy, including regular treatments, such as anti-hypertension, glucose lowering, and serum lipid regulation. Neurological physicians supplied the outpatient service and telephone follow-ups to the patients or their relatives every 3 months over the next year to obtain the recovery information of the post-discharge patients and to determine whether any other clinical cerebrovascular events or deaths occurred. The Modified Rankin Scale (mRS) was utilized to evaluate neurological functional recovery in the 3 months and 1 year. We defined well prognosis as mRS ≤ 2, while bad prognosis was defined when mRS >2 (Turc et al., 2015). However, the clinical cerebrovascular events were defined as the onset of diseases, which forced the patients to go to hospital and undergo image examinations and treatments of the nervous system (Kang et al., 2012). Vascular deaths were defined as sudden death (including cardiac death, death caused by recurrent cerebral infarction or ICH) but not related to tumors, infection or suicide.

### Data analysis

Statistical analysis was performed using SPSS18.0 (SPSS Inc., Chicago, IL, USA) and GraphPad Prism 5.0 (GraphPad Software, San Diego, CA, USA). Baseline clinical data and imaging data were compared with or without CMBs. Continuous variables were expressed as the mean ± *SD* or median (interquartile range). Comparisons were evaluated using Student's *t*-test or the Mann Whitney *U*-test according to whether the data fit a normal distribution. However, continuous variables were expressed the percentage and were compared using χ^2^ or Fisher's exact tests. Backward stepwise multiple logistic regression analysis was performed to analyse the relationship between neurological functional recovery in 3 months after onset and CMB number, as well as the CMB independent risk factors. The Spearman rank correlation coefficient was determined to assess the relationship between CMB number and haematoma volume. The Cox proportional hazards model was established to calculate the risk ratio of clinical cerebrovascular events or vascular deaths in the 1-year follow-up. Corrections of the model included the age, gender, atrial fibrillation, stroke history, haematoma volume, CMBs, SBI, leukoaraiosis, and other factors. *P* < 0.05 was considered a statistically significant difference.

## Results

### General data

Of 130 patients screened, 30 among them were excluded for the reasons below: 11 patients for more than 3 days from onset, 8 for cerebral amyloid angiopathy based on the Boston diagnostic criteria, 5 for trauma, 3 for tumors, 2 for rejection of participation and 1 for the contraindication of MRI. Thus, a total of 100 patients participated in this study. Fifty-five patients (55%) were diagnosed with CMBs, consisting of 37 males (67.3%) and 18 females (32.7%) with an average age of 63 ± 14. Moreover, 45 patients (45%) had no CMBs, and 28 males (62.2%) demonstrated an average age of 58 ± 11. All subjects exhibited hypertension histories ranging from 0.3 to 30 years. The time between disease onset and CT examination ranged from below 24 h (85 patients), 24~48 h (11), and 48~72 h (4). Cranial MRI was provided to the patients on the fifth day after onset. Ninety-eight patients received the follow-up, and during the 3 months from onset, 82 patients had good prognosis (83.7%).

### Affecting factors of CMBs

The comparisons at baseline between patients with or without CMBs are shown in Table 1. Compared with patients without CMBs, positive patients demonstrated increased blood urea nitrogen, creatinine, homocysteine levels, and the prevalence of leukoaraiosis (*P* = 0.038, *P* = 0.04, *P* = 0.001, *P* = 0.009). When establishing backward stepwise multiple logistic regression models, it was also found that patients who were of old age (odds ratio [OR] = 1.04; 95% confidence interval [CI], 1.00–1.08; *P* = 0.039) or had increased serum creatinine levels (OR = 1.03; 95% CI, 1.00–1.05; *P* = 0.011) or leukoaraiosis (OR = 2.97; 95% CI, 1.28–6.89; *P* = 0.016) were more likely to suffer from CMBs.

### CMBs and SBI

Eleven HICH (11%) patients demonstrated SBI in DWI sequences with a total SBI number of 14. SBI was round or ovoid, and all of the foci were very small (diameters ranging from 2.5 to 14.4 mm). In addition, SBI was not relevant with the clinical symptoms, signs, and disease progression. Among the SBI patients, seven occurred in the basal ganglia area, while three occurred in the lobes, and 1 was found in the brainstem. Among the SBI patients, 10 had CMBs (90.1%), and these patients with CMBs also had a predisposition for SBI (18.2 vs. 2.2%, *P* = 0.027).

### CMBs and ICH

In 55 patients with CMBs, we found a total of 431 CMBs, of which 287 (66.6%) were located in the basal ganglia and thalamus, 92 (21.3%) were located in the subcortical area, 38 (8.8%) were located in the brainstem, and 14 (3.2%) were located in the cerebellum.

**Figure 1:**
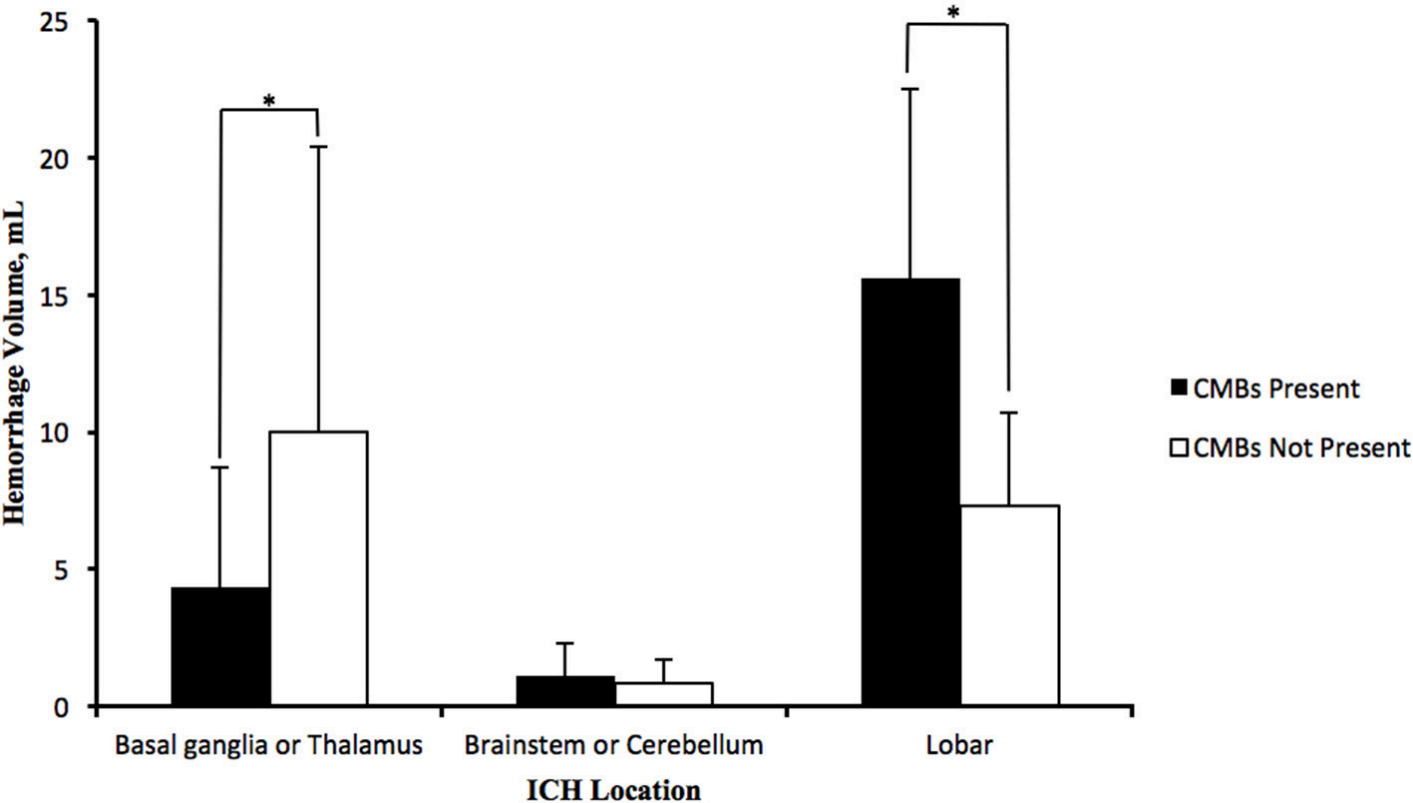
**Distribution of average haematoma volumes of HICH in deep brain (basal ganglia and thalamus), infratentorial part (brainstem and cerebellum) and lobes**. Lobe hemorrhage patients with CMBs possessed more haematoma volumes than patients without CMBs; deep brain hemorrhage patients with CMBs, however, possessed less haematoma volumes than those without CMBs, and the haematoma volumes in infratentorial hemorrhage patients with CMBs were nearly equivalent to patients without CMBs. The data in the graph were expressed in the form of the average ± standard deviation (^*^P < 0.05).

In patients with lobe hemorrhage, higher haematoma volumes were discovered in patients with CMBs (15.60 ± 6.93 vs. 7.33 ± 3.39 mL; *P* = 0.002), and a positive correlation was observed between CMB number and haematoma volume (*r* = 0.677, *P* = 0.006). However, in the patients with deep brain hemorrhage (basal ganglia and thalamus), higher haematoma volumes were found in patients without CMBs (4.37 ± 4.33 mL vs. 10.03 ± 10.38 mL; *p* = 0.005) with a negative correlation between the CMB number and haematoma volumes (*r* = −0.243, *P* = 0.015). For infratentorial hemorrhage patients, no significant differences in haematoma volumes were observed between the groups with or without CMBs (Figure 1).

### CMBs and prognosis

The ratio of poor 3-month prognosis in HICH patients with CMBs was higher than that in patients without CMBs (81.3 vs. 51.2%, *P* = 0.026). Univariate analysis revealed a poorer prognosis in patients with CMBs ≥5 than patients without CMBs (30.8 vs. 7.0%; OR = 11.28; 95% CI, 2.72–46.76; *P* < 0.001). Backward stepwise multiple logistic regression analysis also indicated that CMBs ≥5 were one of the independent risk factors for poor prognosis in 3 months (OR = 27.09; 95% CI, 3.75–195.60; *P* = 0.001, Table 2).

**Table 2:** **Correlation analysis of CMBs number and 3-month functional recoveries of patients**.

	**Unfavorable outcome, 3-month mRS score** >**2**					
	**Univariable analysis**			**Multivariable analysis**		
	**OR**	**95% CI**	***P*-value**	**OR**	**95% CI**	***P*-value**
No CMB	1 (reference)	1 (reference)	1 (reference)		1 (reference)	1 (reference)
1 CMB	2.67	0.23–30.80	0.432	10.34	0.62–172.14	0.103
2–4 CMBs	0.56	0.06–5.65	0.619	0.65	0.04–12.04	0.775
≥5 CMBs	11.28	2.72–46.76	<0.001	27.09	3.75–195.60	0.001

**Figure 2:**
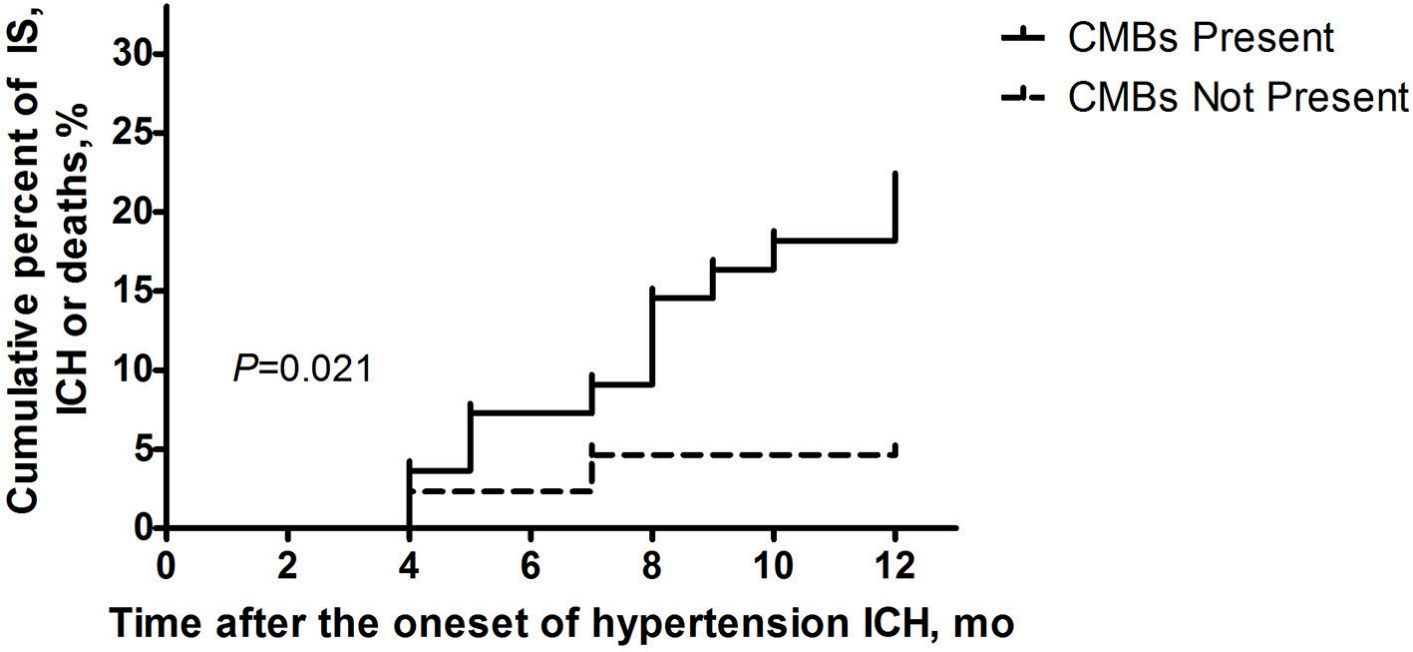
**Ratio of clinical cerebrovascular events or vascular deaths 1 year after disease onsets in the two groups of patients (with/without CMBs) using Kaplan-Meier analysis**. Log-rank examination showed the existence of significant differences between the curves of two groups (*P* = 0.021).

**Figure 3:**
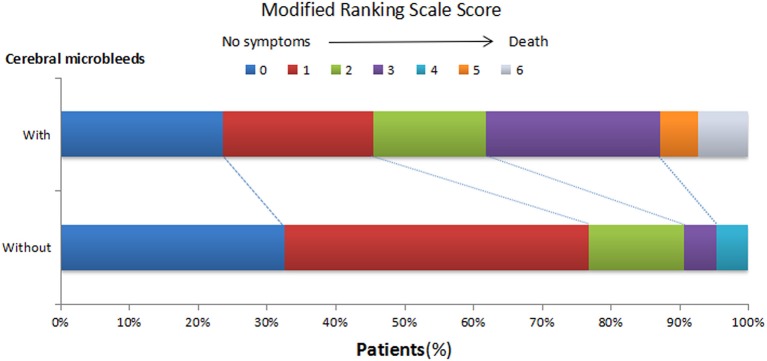
**Distribution of modified Ranking Scale (mRS) score in 1-year follow-ups in HICH patients with or without CMBs**. At 1 year after the onset, the mRS scores of patients with CMBs shifted upwards.

Fourteen patients presented with cerebrovascular events or deaths during the 1 year follow-up, which consisted of nine cerebral infarctions, one recurrent cerebral hemorrhage and four vascular deaths (Figure 2). In those 14 patients, 6 were with SBI (42.86%), and 12 were with CMBs (85.71%, 11 with CMBs ≥5). Moreover, in these 12 patients, 7 presented with recurrent cerebral infarction, and 1 demonstrated recurrent cerebral hemorrhage. The number of recurrent cerebral infarction patients with both SBI and CMBs was 5. Patients with CMBs had a higher mRS score in the first year than those without CMBs (Figure 3). The multifactorial Cox proportion analysis model showed that atrial fibrillation, CMBs ≥5 and with SBI were independent predictive factors for the occurrence of cerebrovascular events or vascular deaths in 1 year from onset.

## Discussion

We concluded the following results: (1) backward stepwise multiple logistic regression analysis indicated that old age, elevation of serum creatinine level and complicated with leukoaraiosis were independent risk factors of CMBs; (2) the prevalence of SBI was highest in HICH patients with CMBs; (3) the existence and number of CMBs were relevant with haematoma volumes in the lobes and deep brain; and (4) poorer neurological functional recoveries in 3 months from onset were observed in patients with CMBs ≥5 than those without CMBs. In addition, atrial fibrillation, CMBs ≥5 and SBI were observed as independent predictive factors in clinical cerebrovascular events or vascular deaths.

The prevalence of CMBs in primary or secondary intracerebral hemorrhage patients is 50~80% (Gregoire et al., 2010). We discovered that the prevalence of CMBs in HICH patients was 55%, and old age, elevation of serum creatinine level and with leukoaraiosis played a role in independent risk factors in CMB onset, which corresponded with previous research studies (Poels et al., 2010; Ryu et al., 2012; Yamada et al., 2012; Akoudad et al., 2013). Targeting the community population, Poels et al., discovered that the prevalence of CMBs increased with age. For instance, the prevalence of CMBs in a population aged over 80 years was five times more than that in the population aged between 45 and 50 years (Poels et al., 2010). Ryu et al. found that among 909 cerebral infarction patients, chronic kidney disease was independently relevant with CMBs (Ryu et al., 2012). In addition, Yamada et al. revealed a close relationship between the number of CMBs and periventricular hyperintensity (PVH) or deep white matter hyperintensity (DWMH) and regarded leukoaraiosis as an independent risk factor of CMB severity (Yamada et al., 2012). In addition, in DWI sequences, we found that approximately 11% of HICH patients suffered from acute infarction in which the incidence was even higher in patients with CMBs. Numerous recent research studies have reported the existence of SBI in DWI sequences of ICH patients (Okamoto et al., 2010; Prabhakaran et al., 2010; Kang et al., 2012; Arsava et al., 2013). In addition, most of these research studies showed that the onset of post-ICH DWI foci was correlated with over acute decline of blood pressure, but the underlying mechanism was still unclear. The CMBs, SBI and leukoaraiosis all belonged to CSVD. The research studies of Wardlaw et al. illustrated that CSVD was caused by brain tissue hypoperfusion and brain parenchyma damage induced by blood brain barrier destruction after vascular endothelial injury and, interestingly, the various subtypes of interconnected CSVD (Wardlaw et al., 2013a). However, due to the insidious and undistinguishable onset, the clinical symptoms of CSVD could only occur after a long progression. In addition, due to hardness on the small vessels imaging, CSVD was then easily ignored (Wardlaw et al., 2001).

CMBs are a type of intracerebral micro lesion with hemorrhage tendency (Nighoghossian et al., 2002), and thus when presented with CMBs, the probability of hemorrhage as well as the haematoma volumes in patients are increased. Lee et al. reported that lobe and putamen hemorrhage patients with CMBs exhibited higher haematoma volumes than those without CMBs and together present a positive correlation relationship between haematoma volumes and CMB number (Lee et al., 2006). Nevertheless, the haematoma volumes of thalamus hemorrhage patients were not relevant to CMBs. They proposed that CMBs might reflect the damaging degree of the blood brain barrier. Thus, lobe and putamen hemorrhage patients with CMBs had more haematoma volumes. As for the thalamus, the hemorrhage might be restricted by surrounding tissues, and the frequency of intraventricular hemorrhage was higher, which resulted in decreased bleeding volumes. In our study, the results in lobe hemorrhage patients corresponded with those in Lee's research, and the haematoma volumes in patients with CMBs were nearly twice as much as those in patients without CMBs. However, in contrast with Lee's reports, we found decreased haematoma volumes in basal ganglia or thalamus hemorrhage patients with CMBs and a negative correlation relationship between haematoma volumes and the number of CMBs. This difference might be due to the design of the study, the groups recruited, the time between disease onset and CT examination, the methods to measure the haematoma volumes and the differences in MRI parameters. Thus, the effect on haematoma volumes in different regions of CMBs deserves further study.

CMBs may increase the incidence of primary or secondary stroke and even mortality in patients (Nighoghossian et al., 2002; Greenberg et al., 2004). In a population aged 70–82, the risk of stroke related deaths in patients with CMBs ≥2 was six times higher than that in patients without CMBs. When CMBs are located in non-lobe regions, the risk of future cardiac death was twice as high as those without CMBs (Altmann-Schneider et al., 2011). However, differences were present regarding the effects of CMBs on the 3-month functional prognosis after intravenous thrombolysis in patients with acute ischaemic stroke (Gratz et al., 2014; Turc et al., 2015; Yan et al., 2015). However, few studies have been published examining the relationship between CMB number and HICH prognosis. In addition, our study found a close relevance between CMBs ≥5. With regard to pathophysiology, CMBs reflected the bleeding tendency of the brain, but during the 1-year follow-ups, we discovered that the recurrent stroke in patients with CMBs were mostly due to cerebral infarctions rather than ICH. Recently, controversies have arisen indicating that patients with CMBs might demonstrate an increase on the following ICH (Linn, 2015). In addition, some research studies showed that in patients with cerebral amyloid angiopathy, only patients with CMBs located in the lobes (specifically CMBs ≥5) suffered from an increasing risk of future ICH (Biffi et al., 2010). However, in our study, CMBs were mostly located in the basal ganglia and thalamus. Thus, the location of the CMBs may be potentially relevant in determining whether CMBs could help predict the risk of future cerebral stroke.

However, there were some shortcomings in this study: (1) The information of patients suffering from ICH was not blinded to the MRI readers. To reduce the deviations in CMBs quantification, the radiologists did not know our hypothesis and the relationship between the ICH and CMBs. In addition, the measurements for haematoma volumes and CMBs number were performed independently. (2) We excluded ICH patients caused by cerebral amyloid angiopathy according to the Boston criteria and selected patients with a history of hypertension. However, no brain pathological examinations were performed, and our subjects might thus be complicated with cerebral amyloid angiopathy, which might affect the results. (3) Due to the rigidity of the inclusion criteria, the number of enrolled patients was limited, and further research studies with more cases included are needed.

## Conclusion

Overall, we proposed that elderly subjects, those with increased serum creatinine levels and leukoaraiosis are independent risk factors for the incidence of CMBs; furthermore, CMB patients had a higher probability when presented with SBI. CMBs ≥5 affected neurological functional recoveries within 3 months after disease onset in HICH patients. Patients with CMBs ≥5 or SBI might demonstrate increasing risks for clinical cerebrovascular events or vascular deaths in 1 year. In the future, if adequate attention and intervention is provided to patients with CMBs proven by a SWI sequence but without ICH, the incidence and bleeding volume of future ICH may be reduced.

## Author contributions

KY, designed the study, analyzed and interpreted the data in study, drafted and revised the manuscript; YuF, designed the study, drafted and revised the manuscript; JM, drafted and revised the manuscript, acquisition of data; NF, drafted and revised the manuscript, acquisition of data; SC, study concept and design, revised the manuscript, English language editing; YiF, study concept and design, revised the manuscript, supervised, and coordinated the study. All authors have read and approved the final manuscript.

## Conflict of interest statement

The authors declare that the research was conducted in the absence of any commercial or financial relationships that could be construed as a potential conflict of interest. The reviewer GY declared a shared affiliation, though no other collaboration, with several of the authors KY, YF, NF to the handling Editor, who ensured that the process nevertheless met the standards of a fair and objective review.
